# Interplay Between Thiamine and p53/p21 Axes Affects Antiproliferative Action of Cisplatin in Lung Adenocarcinoma Cells by Changing Metabolism of 2-Oxoglutarate/Glutamate

**DOI:** 10.3389/fgene.2021.658446

**Published:** 2021-04-01

**Authors:** Vasily A. Aleshin, Xiaoshan Zhou, Shuba Krishnan, Anna Karlsson, Victoria I. Bunik

**Affiliations:** ^1^Faculty of Bioengineering and Bioinformatics, Lomonosov Moscow State University, Moscow, Russia; ^2^Belozersky Institute of Physico-Chemical Biology, Lomonosov Moscow State University, Moscow, Russia; ^3^Division of Clinical Microbiology, Department of Laboratory Medicine, Karolinska University Hospital, Karolinska Institute, Stockholm, Sweden; ^4^Department of Biological Chemistry, Sechenov University, Moscow, Russia

**Keywords:** p21, p53, cisplatin, thiamine deficiency, TCA cycle, glutamate metabolism, A549 adenocarcinoma cells

## Abstract

Thiamine (vitamin B1) is often deficient in oncopatients, particularly those undergoing chemotherapy. However, interaction between the thiamine deficiency and anticancer action of drugs has not been characterized. A major natural thiamine derivative, thiamine diphosphate (ThDP), is a coenzyme of central metabolism, also known to affect transcriptional activity of the master metabolic regulator and genome guardian p53. A direct transcriptional target of p53, p21, regulates cell cycle dynamics and DNA damage response. Our work focuses on dependence of the action of the DNA damaging anticancer drug cisplatin on metabolic regulation through p53/p21 axes and cellular thiamine status in human lung adenocarcinoma cells A549. These cells are used as a model of a hardly curable cancer, known to develop chemoresistance to platinum drugs, such as cisplatin. Compared to wild type (A549^WT^), a stable line with a 60% knockdown of p21 (A549^p21–^) is less sensitive to antiproliferative action of cisplatin. In contrast, in the thiamine-deficient medium, cisplatin impairs the viability of A549^p21–^ cells more than that of A549^WT^ cells. Analysis of the associated metabolic changes in the cells indicates that (i) p21 knockdown restricts the production of 2-oxoglutarate *via* glutamate oxidation, stimulating that within the tricarboxylic acid (TCA) cycle; (ii) cellular cisplatin sensitivity is associated with a 4-fold upregulation of glutamic-oxaloacetic transaminase (GOT2) by cisplatin; (iii) cellular cisplatin resistance is associated with a 2-fold upregulation of p53 by cisplatin. Correlation analysis of the p53 expression and enzymatic activities upon variations in cellular thiamine/ThDP levels indicates that p21 knockdown substitutes positive correlation of the p53 expression with the activity of 2-oxoglutarate dehydrogenase complex (OGDHC) for that with the activity of glutamate dehydrogenase (GDH). The knockdown also changes correlations of the levels of OGDHC, GDH and GOT2 with those of the malate and isocitrate dehydrogenases. Thus, a p53/p21-dependent change in partitioning of the glutamate conversion to 2-oxoglutarate through GOT2 or GDH, linked to NAD(P)-dependent metabolism of 2-oxoglutarate in affiliated pathways, adapts A549 cells to thiamine deficiency or cisplatin treatment. Cellular thiamine deficiency may interfere with antiproliferative action of cisplatin due to their common modulation of the p53/p21-dependent metabolic switch between the glutamate oxidation and transamination.

## Introduction

Cisplatin and similar platinum complexes, which remain the first line anticancer drugs, are known inducers of DNA damage. Proliferation and DNA damage response of cancer cells with functional p53 pathway is controlled by p21, an inhibitor of the cyclin-dependent kinase involved in cell cycle, and a direct transcriptional target of p53 (Sarin et al., 2017; Guo et al., 2018). Accordingly, the anticancer action of cisplatin is mediated by p53/p21-dependent cell cycle arrest and apoptosis (Qu et al., 2013; Sarin et al., 2017; Deng et al., 2019; Xu et al., 2019a), which may also be observed with other drugs, such as oxfendazole acting in non-small cell lung cancer (Xu et al., 2019a). However, this relationship is rather conditional, strongly affected by different factors. Dependent on the cancer type, p21 may slow down tumorigenesis, or support tumor growth by slowing down the accumulation of DNA damage (Shamloo and Usluer, 2019). In the cisplatin-sensitive and cisplatin-resistant cells up-regulation of p21 and its cytosolic translocation have different consequences for the cisplatin antiproliferative effect (Xia et al., 2011; Lu et al., 2016; Sarin et al., 2017). In ovarian cancer, p53/p21pathway mediates antiproliferative action of cisplatin, but simultaneously cisplatin induces a p53-associated cellular protein involved in the cisplatin resistance (Yang et al., 2019). Thus, the DNA damage response elicited through the p53/p21axes may depend on the metabolic and regulatory features of different cancer cells, tightly linked to development of chemoresistant cells through the complex and fragmentary characterized network of regulatory interactions associated with p53/p21pathway.

In addition to cell damage through DNA binding, the platinum complexes provide their anticancer action affecting metabolism (Zhu et al., 2019). Cellular regulation, such as the p53-dependent one, must support cellular homeostasis, and therefore is always involved with metabolism. Cellular decisions on death or survival may be better deduced from different metabolic patterns rather than different expression of regulatory proteins, as not only the expression, but also a multitude of post-translational modifications and incorporation into different supramolecular structures affect functional outcome of the regulatory protein expression. For instance, like p21 expression, mitochondrial changes are also known to contribute both to the antiproliferative action of cisplatin (Zhu et al., 2019) and to the development of resistance of cancer cells to the cisplatin action (Cruz-Bermudez et al., 2019). However, the anticancer action of the platinum drugs involves inhibition of both oxidative phosphorylation and glycolysis (Zhu et al., 2019), whereas the resistance to cisplatin is observed upon increased mitochondrial oxidative phosphorylation along with decreased glycolysis (Cruz-Bermudez et al., 2019). Thus, metabolic adaptations may underlie the observations of opposite outcomes of p21 expression for the cisplatin antiproliferative action.

Considering the metabolic aspect of the complex relationship between the cisplatin action and p21 expression, it is worth noting that not only accumulating mutations, but also availability of nutrients and vitamins, may change cancer metabolism with tumor progression and chemotherapy. In A549 cells, metabolic impairments upon knockouts of aspartate aminotransferase GOT1 or dicarboxylate transporter SLC25A10 induce downregulation of p21 (Zhou et al., 2015, 2018). Metabolism of dicarboxylates is tightly linked to the tricarboxylic (TCA) cycle, whose rate-limiting reaction, particularly in A549 cells, is catalyzed by a multienzyme mitochondrial complex of 2-oxoglutarate dehydrogenase (OGDHC) (Bunik et al., 2016). Functional importance of OGDHC in these cells is linked to their strong dependence on the dicarboxylate transporter SLC25A10 and the mitochondrial part of the malate-aspartate shuttle involving aminotransferase GOT2 (Bunik et al., 2015, 2016; Allen et al., 2016; Bunik, 2017; Anderson et al., 2018). On the other hand, p21 knockdown in A549 cells dysregulates saturation of OGDHC with its coenzyme—diphosphorylated thiamine (vitamin B1), associated with the opposite actions of the coenzyme deficiency or excess in the lung adenocarcinoma and normal epithelium (Bunik et al., 2020b). Our recent studies of the relation between the thiamine-dependent processes in A549 cells, p53/p21 axes and cellular response to cisplatin (Bunik et al.,2020a,b) indicate significant changes in these processes after cisplatin exposure, suggesting thiamine involvement with cisplatin effects. It is important to note in this regard that many oncopatients have subclinical (without classic symptoms) thiamine deficiency, which may increase as a result of anticancer therapies (Ahmad et al., 2016; Choi et al., 2016; Isenberg-Grzeda et al., 2016, 2017; Onishi et al., 2018; Yoshioka et al., 2020; Zhang et al., 2020). However, the role of thiamine deficiency and thiamine supplementation during the anticancer treatments is practically not investigated. A study shows that in a subset of leukemia cells, increasing thiamine elevates the sensitivity to anticancer treatments with asparaginase (Guarecuco et al., 2020). Thiamin is also shown to increase sensitivity of breast cancer cells to radiotherapy in a p53/p21-dependent way (McLure et al., 2004). Simultaneous delivery of anticancer drugs and thiamine increased the antiproliferative effect (Singh et al., 2016). However, many studies warn that the vitamin-rich nutrition may decrease the anticancer effect of cisplatin, e.g., through the pyridoxal, thiamine and riboflavin competition for cisplatin binding to DNA (Szefler et al., 2019). It is worth noting in this regard that such alerts stem from a well-known antioxidant action of thiamine in normal cells rather than in cancer ones. Indeed, in non-malignant cells exposed to drugs including cisplatin, and other pathological conditions, such as diabetes, ischemia-reperfusion, or hemodialysis, thiamine and/or its derivatives are known to decrease DNA damage and cell death (Schupp et al., 2008; Chakrabarti et al., 2011; Coskun et al., 2013; Harisa, 2013; Suleyman et al., 2013; Turan et al.,2013a,b; Vidhya et al., 2013; Cinici et al., 2015; Polat et al., 2015). A recent transcriptional analysis of long-term exposure of yeast cells to anticancer drug imatinib supports contribution of biosynthesis of thiamine/pyridoxal phosphate to the drug resistance in this model (Taymaz-Nikerel et al., 2020). Yet studies of malignant cells indicate that the effects of thiamine supplementation (reviewed in Bunik et al., 2020a), or of targeting thiamine metabolism (Tiwana et al., 2015; Yang et al., 2016) strongly differ in the cancer and normal cells. Obviously, the well-known difference in metabolism and its regulation in these cells reciprocates their different reactions to changes in cellular thiamine status, defined by the levels of different *in vivo* derivatives of thiamine and their metabolic interconversions.

Metabolic effects of thiamine are based on the coenzyme action of its diphosphate ThDP in the cytosolic production of reducing equivalents and ribose through pentose phosphate shunt involving ThDP-dependent transketolase, and in mitochondrial ThDP-dependent reactions catalyzed by 2-oxo acid dehydrogenases. Besides, thiamine and its derivatives may regulate metabolism also by their non-coenzyme action, exemplified by the ThDP interaction with p53 (McLure et al., 2004; Aleshin et al., 2019). The goal of this work is to establish if/how the antiproliferative action of cisplatin depends on the thiamine status of the lung adenocarcinoma cells. By screening key reactions of energy metabolism, potentially regulated by thiamine and p53/p21 axes (Bunik et al., 2020a), we reveal specific metabolic switch between the glutamate oxidation and transamination, which is involved with cellular response to cisplatin and regulated by the p53/p21 axes and thiamine status in a highly interactive manner.

## Materials and Methods

### Cells, Cultivation, and Reagents

Human epithelial adenocarcinoma non-small cell lung cancer line A549 (ATCC^®^ CCL-185^TM^) was used. Construction of the stable p21 knockdown A549 cells (A549^p21–^) was described previously (Bunik et al., 2020b). The knock-down and mock control cells were maintained with puromycin at concentration of 0.2 μg/ml. The cells were cultivated in Dulbecco’s Modified Eagle’s medium (DMEM) (Thermo Fischer Scientific, 21855), which comprised 1 g/L glucose, 1 mM pyruvate, 4 mM GlutaMAX^TM^, 10% FBS (Gibco, 10270), 100 U/ml penicillin and 0.1 mg/ml streptomycin (Thermo Fisher Scientific, 15140), at 37°C, 5% CO_2_ in a humidified atmosphere.

The protease and phosphatase inhibitor cocktails cOmplete^TM^ (Roche, 04693116001) and PhosSTOP^TM^ (Roche, PHOSS-RO) were used. Cisplatin (PHR1624), NAD^+^ (N7004), NADP^+^ (N5755), isocitrate (I1252), nicotinamide (72340), ThDP (C8754), NADH (N8129), malic acid (240176), oxaloacetate (O4126), 2-oxoglutarate (K1750), CoA (C4282), sodium deoxycholate (D6750), IGEPAL CA-630 (I8896) were from Merck (Sigma-Aldrich).

### Cell Counting, Diameter Evaluation, and Imaging

The cell diameter evaluation and cell counting were performed using mini automated cell counter (Moxi, Orflo Technologies). Twenty-five thousand cells were seeded into 6-well plates and the number and diameter of cells were analyzed 2, 4, and 6 days later, while the cells were in the log growth phase. The images of the A549^WT^ and A549^p21–^ cells were obtained at day 6 using an optical microscope with the camera adaptor.

### Thiamine Deficiency Cell Model

Establishment of the thiamine deficient cells was described before (Bunik et al., 2020b). Briefly, thiamine-free home-made DMEM, analogous to the medium used for the cell growth, and dialyzed 10% FBS (Sigma, F0392) were used. The cells were made thiamine deficient by their plating into the thiamine-free DMEM, followed by the 7 days growth in this medium. During this period, the cells were reseeded into two flasks on the fourth day using fresh thiamine-free DMEM.

### Supplementation of Cisplatin or ThDP and Preparation of Cell Lysates

2 × 10^5^ A549 cells were seeded in each well of 6-well plates and attached during 24 h. Next day the medium was replaced by the same fresh medium or the medium supplemented with 5 μM cisplatin or 5 mM ThDP, followed by the 24 h incubation period. During this period cells could grow to no more than 80% confluency, staying in the log growth phase. Then, they were washed with PBS twice and lysed (30 min of slow shaking on ice) in radioimmune precipitation assay buffer (50 mM HEPES, pH 7.5, 150 mM NaCl, 1% IGEPAL CA-630, 0.05% sodium deoxycholate, and the protease and phosphatase inhibitor cocktails). The lysed cells were scraped prior to collection of lysates.

### Evaluation of GOT2, p53 and p21 Expression

The levels of GOT2, p53 and p21 were analyzed as described previously (Bunik et al., 2020a). Briefly, the p21 mRNA expression was analyzed by real-time PCR (Kapa Biosys-tems, Wilmington, MA). RNeasy mini kit (74106, QIAGEN) was used for RNA preparation. Beta-actin and S18 mRNAs were used as loading controls. The GOT2 and p53 proteins were evaluated by western blotting using anti-GOT2 (Sigma, HPA018139) and anti-p53 (Santa Cruz Bio-technology, sc-126) primary antibodies, with the anti-β-actin (Sigma, A5441) and anti-VDAC/porin (Cell Signaling Technology, 4661) antibodies used as loading controls. The protein expression levels were quantified with ImageJ2 (Schindelin et al., 2015).

### Cell Viability Assay

Cellular viability was determined using a Cell Proliferation Kit II (Roche, 11465015001) as described before (Bunik et al., 2020b).

### Enzyme Activity Assays

The OGDHC activity was assayed at endogenous, i.e., present in cellular lysates, ThDP level, or with 1 mM ThDP added to the assay medium. The former assay estimates intracellular portion of active OGDHC, i.e., the level of the holoenzyme with bound ThDP (holo-OGDHC). The latter assay estimates total amount of both the active (holoenzyme) and latent (apoenzyme) forms of OGDHC. Besides, the total levels of GDH, MDH, NADP^+^-dependent malic enzyme, NADP^+^-dependent IDH, NAD^+^-dependent IDH were measured as described earlier (Bunik et al., 2020a). The enzymatic activities were normalized to total protein content, determined with Bio-Rad Protein Assay Kit I (Bio-Rad, 5000001), and expressed as micromoles of the consumed substrate or generated product per min per mg of protein.

### Statistical Analysis

Statistical analysis was performed using GraphPad Prism, version 7.0 (GraphPad Software Inc., La Jolla, United States). To apply ANOVA and also Pearson criterion for the correlation analysis, Gaussian distribution of the parameters was checked by the D’Agostino & Pearson omnibus normality test. Comparisons between multiple experimental groups were done using two-way ANOVA with *post hoc* Tukey’s test. Mann-Whitney test was used for comparison of two groups. Significance of the differences between groups is indicated on the graphs according to the following code: ^****^ – *p* < 0.0001; ^∗∗∗^ – 0.0001 < *p* < 0.001; ^∗∗^ – 0.001 < *p* < 0.01; ^∗^ – 0.01 < *p* < 0.05; # – 0.05 < *p* < 0.1.

## Results

### Morphological and Functional Changes in A549 Cells With Knockdown of p21

**FIGURE 1:**
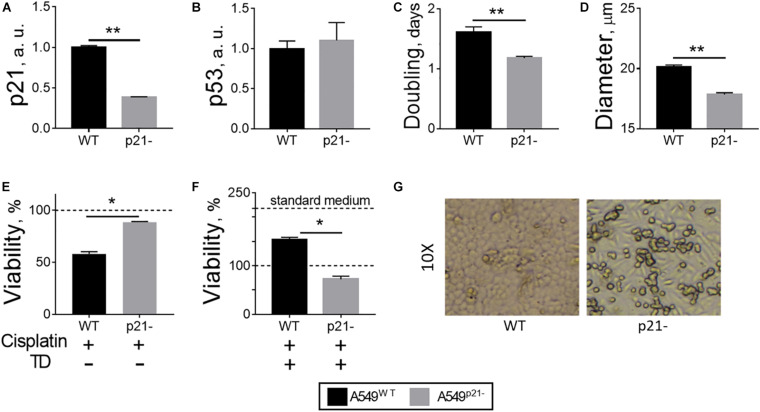
Changes in A549 cells, observed upon p21 knockdown. **(A)** p21 mRNA transcripts. **(B)** p53 protein. **(C)** Doubling time during growth in culture in log phase. **(D)** Diameter of detached cells. **(E)** Cellular viability after 24 h incubation of the cells with 5 μM cisplatin in standard medium. **(F)** Cellular viability after 24 h incubation of the cells with 5 μM cisplatin in thiamine-deficient (TD) medium. **(G)** Morphology of A549^WT^ and A549^p21–^ cells grown in standard medium for 6 days, shown at a 10-fold magnification. In **(A,B)**, a.u. define arbitrary units of expression. In **(E,F)**, the viability is assessed by cellular NAD(P)H:XTT reductase activity and normalized to control values without cisplatin in the standard **(E)** and thiamine-deficient **(F)** media. In view of a 2-fold decrease of the viability by thiamine deficiency, 100% NAD(P)H:XTT reductase activity in **(E)** corresponds to 218% of NAD(P)H:XTT reductase activity in **(F)**, shown by additional dash line “standard medium.” The data are averaged from the three independent experiments. Values are presented as mean ± SEM. Statistically significant differences are determined according to the Mann-Whitney test. Different *p*-values are shown by asterisks as described in Materials and Methods under Statistical Analysis section.

General properties of the constitutive p21 knockdown A549 cell line (A549^p21–^) with a 60% decrease in p21 expression (Figure 1A) have been compared to the original A549 cell line (A549^WT^). The knockdown and wild type cells possess the same level of p53, when cultured in standard media, indicating that the decrease in p21 level does not affect expression of its transcriptional regulator—the p53 protein (Figure 1B). A549^p21–^ cells grow 25% faster than A549^WT^ cells (Figure 1C), while their size is smaller, manifested in a 12% decrease in the diameter of the cells detached from the surface for counting (Figure 1D). This change in diameter equals to a 30% decrease in cell volume and more than 20% decrease in cell surface area, taking into account that the detached cells have spherical form.

In agreement with the known requirement of p21 for the cisplatin anticancer action (Shamloo and Usluer, 2019), the viability of A549^p21–^ cells is decreased by 5 μM cisplatin only by 11 ± 1%, whereas the viability of A549^WT^ under the same conditions is decreased by 42 ± 2% (Figure 1E). However, this “protective” effect of p21 knockdown is not observed in the thiamine-free medium (Figure 1F), where the viability of thiamine-deficient cells, both A549^WT^ and A549^p21–^, is only about half of that in the thiamine-supplemented medium (Figure 1F, standard medium), as shown by us earlier (Bunik et al., 2020b). That is, when A549 cells are thiamine-deficient, cisplatin decreases the viability by 28 ± 6% only of A549^p21–^ cells. In the thiamine-deficient A549^WT^ cells cisplatin competes with the effect of thiamine deficiency, partially (by 53 ± 6%) restoring the viability lost by A549^WT^ cells due to the thiamine deficiency (Figure 1F). Thus, antiproliferative action of cisplatin in the thiamine-deficient medium adds to that of thiamine deficiency in A549^p21–^ cells, but is competitive to that of thiamine deficiency in A549^WT^ cells. As a result, p21 knockdown decreases the cisplatin sensitivity of A549 cells in the thiamine-supplemented medium, increasing it in the thiamine-free medium.

Remarkably, the shape of attached A549^p21–^ cells demonstrate elongation absent in the A549^WT^ cells (Figure 1G). The morphological change is reminiscent of epithelial-mesenchymal transition, known to reduce cisplatin sensitivity (Deng et al., 2019; Bahar et al., 2020; Brum et al., 2020; Chen et al., 2020; Siraj et al., 2020), which is also observed in our experiments (Figure 1E).

### Metabolism of Glutamate and Related Dicarboxylates Is Strongly Changed by p21 Knockdown in A549 Cells

**FIGURE 2:**
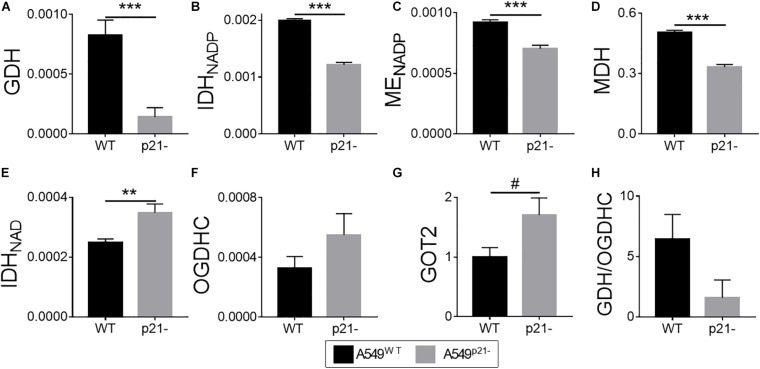
Effect of p21 knockdown in A549 cells on the levels of the TCA cycle and affiliated enzymes. **(A–F)** Activities of the enzymes indicated on Y axes are shown as μmol⋅ min^–1^⋅ mg^–1^; **(G)** protein level of the mitochondrial aspartate aminotransferase (GOT2), arbitrary units; **(H)** ratio of activities of GDH to total OGDHC, assessing the maximal glutamate degradation in the TCA cycle. The data are obtained from three independent cell cultures. Values are presented as mean ± SEM. Statistical significance of the differences is determined according to the Mann-Whitney test. Different *p*-values are shown by asterisks and mesh-sign as described in Materials and Methods under Statistical Analysis section.

The physiological and morphological differences presented in Figure 1 are accompanied by significant metabolic changes shown in Figure 2. The selection of enzymes presented in Figure 2, is based on earlier findings which link the downregulation of p21 with impaired dicarboxylate transport to the mitochondria (Zhou et al., 2015, 2018). Thus, p21 knockdown may affect the malate-aspartate shuttle, dependent on MDH, GOT and irreversible degradation of glutamate/2-oxoglutarate in the TCA cycle.

From the enzymes tested, GDH is affected the most by the p21 knockdown, exhibiting a 6-fold decrease in the activity in A549^p21–^ cells, compared to the A549^WT^ cells (Figure 2A). The decrease in the GDH activity, producing 2-oxoglutarate from glutamate, is accompanied by significant decreases in activities of metabolically related dehydrogenases, such as the 2-oxoglutarate-producing NADP^+^-dependent IDH (encoded by *IDH1* and *IDH2*), and dehydrogenases of malate: decarboxylating NADP^+^-dependent malic enzyme (ME, encoded by *ME1, ME2*, and *ME3*) and NAD^+^-dependent MDH (encoded by *MDH1* and *MDH2*) (Figures 2B–D). The measured activities of MDH, NADP^+^-dependent IDH and ME, representing the sum of those inherent in the mitochondrial and cytosolic isoenzymes, characterize general impact of p21 knockdown on transformation of the corresponding metabolites. The decreases in total MDH and IDH activities upon p21 knockdown indicate that A549^p21–^ cells possess changed communication between the cytosol and mitochondria through malate-aspartate shuttle of reducing equivalents. The shuttle relies on the exchange of malate to 2-oxoglutarate, which is the product of the reactions catalyzed by IDH, GDH and GOT. Our study shows that p21 knockdown strongly changes the pathways producing 2-oxoglutarate not only from isocitrate, but also from glutamate. A decrease in GDH activity in A549^p21–^ cells (Figure 2A) is accompanied by increased (*p* < 0.1) level of mitochondrial aminotransferase GOT2 (Figure 2G), which is another component of the malate-aspartate shuttle.

Contrary to the NADP^+^-dependent IDHs, the activity of the NAD^+^-dependent IDH, which precedes OGDHC in the TCA cycle and produces the OGDHC substrate 2-oxoglutarate within the TCA cycle metabolon, is increased 1.4-fold upon p21 knockdown (Figure 2E).

As a result, our data indicate that p21 knockdown in A549 cells strongly affects the mitochondrial interconversion between 2-oxoglutarate and glutamate, added by changes in the activities of the enzymes producing 2-oxoglutarate from isocitrate and the enzymes of malate metabolism. Overall, the metabolic changes observed in A549^p21–^ cells, indicate that they differ from A549^WT^ cells by the glutamate oxidation in the TCA cycle and the exchange of reducing equivalents between cytosol and mitochondria.

### Glutamate Partitioning Between Oxidation by GDH and Transamination by GOT2 Is Changed After Cisplatin Treatment of A549 Cells in a p21-Dependent Manner

**FIGURE 3:**
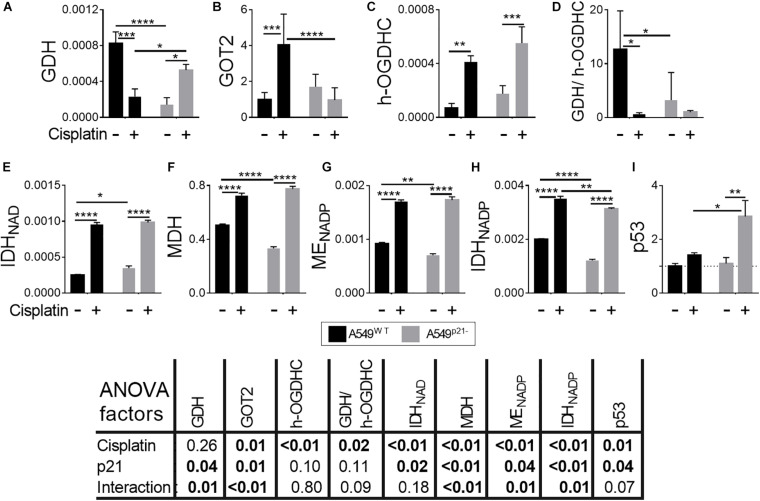
Changes in activities or expression of the enzymes and p53 in A549^WT^ and A549^p21–^ cells induced by cisplatin. **(A,C,E–H)** Activities of the enzymes indicated on Y axes are shown as μmol⋅ min^–1^⋅ mg^–1^; **(B)** protein level of the mitochondrial aspartate aminotransferase (GOT2), arbitrary units; **(D)** ratio of activities of GDH to endogenous holo-OGDHC (h-OGDHC), assessing the glutamate degradation in the TCA cycle; **(I)** protein level of p53, arbitrary units. Where indicated, cells were incubated with 5 μM cisplatin for 24 h. The data are obtained from three independent cell cultures. Values are presented as mean ± SEM. Statistical significance of the differences is determined by two-way ANOVA followed by Tuckey’s *post hoc* test. *P*-values of the significances of the two factors and their interaction, analyzed by ANOVA, are provided in the table under the graphs. On the graphs, different *p*-values are shown by asterisks as described in Materials and Methods under Statistical Analysis section.

Figures 3A–C show how the enzymes involved in the adaptation to p21 knockdown, respond to cisplatin in A549^WT^ and A549^p21–^ cells.

In A549^WT^ cells, 24 h incubation with 5 μM cisplatin upregulates all the studied enzymes but GDH, whose activity is decreased 4-fold (Figure 3). The TCA cycle activation is supported by (i) increased generation of 2-oxoglutarate through the IDH reactions, (ii) a 5-fold increase in the rate-limiting OGDHC holoenzyme, i.e., the active enzyme endogenously saturated with ThDP, and (iii) additional feeding of the TCA cycle by the activated ME producing pyruvate. At the same time, more than a 10-fold drop in GDH/OGDHC ratio ensures that glutamate catabolism is not increased under the metabolic transformation induced by cisplatin. Increases in MDH and GOT2 level favor the malate-aspartate shuttle function and provision of oxaloacetate for the isocitrate synthesis. Overall, the observed enzymatic changes suggest that cisplatin activates the TCA cycle through OGDHC and malate-aspartate shuttle, simultaneously preventing excessive catabolism of glutamate.

In A549^p21–^ cells, 24 h incubation with 5 μM cisplatin upregulates all the studied enzymes, but GOT2 (Figure 3). As the activities of both GDH and holoenzyme of OGDHC are increased 4-fold and 3-fold, respectively, the GDH/OGDHC ratio remains at approximately the same level in the cisplatin-treated A549^p21–^ cells (Figure 3D). Under this condition, the lower level of GOT2 in the cisplatin-treated A549^p21–^ vs. A549^WT^ cells (Figure 3B) should increase the glutamate oxidation in the TCA cycle in A549^p21–^ vs. A549^WT^. Apart from the glutamate metabolism changing differently in A549^p21–^ and A549^WT^ cells after the cisplatin treatment, the treatment abrogates the p21-induced changes in the NAD^+^-dependent IDH, MDH, and ME (Figures 3E–G), and attenuates the difference in the NADP^+^-dependent IDH activity. Finally, cisplatin exposure causes a 3-fold induction of p53 in A549^p21–^ cells, not observed in A549^WT^ cells (Figure 3I). Thus, the higher cisplatin resistance of A549p^21–^ cells, compared to A549^WT^ cells, is associated with the cisplatin-caused induction of p53 and elevated glutamate flux to TCA cycle.

### Metabolic Changes Induced by Thiamine Deficiency in A549^WT^ and A549^p21–^ Cells

**FIGURE 4:**
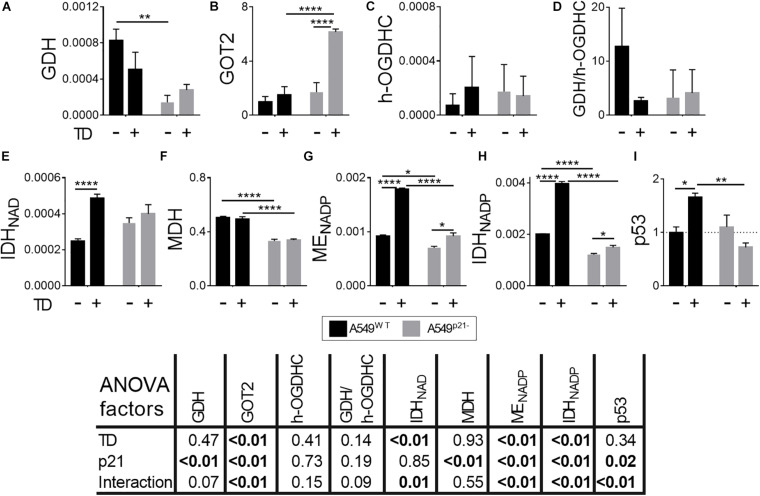
Changes in the levels of the enzymes and p53 in A549^WT^ and A549^p21–^ cells, induced by thiamine deficiency. **(A,C,E–H)** Activities of the enzymes indicated on Y axes are shown as μmol⋅ min^–1^⋅ mg^–1^; **(B)** protein level of the mitochondrial aspartate aminotransferase (GOT2), arbitrary units; **(D)** ratio of activities of GDH and endogenous holo-OGDHC (h-OGDHC), assessing the glutamate degradation in the TCA cycle; **(I)** protein level of p53, arbitrary units. Thiamine deficient (TD) cells were grown for 7 days in thiamine-free medium. The data are obtained from three independent cell cultures. Values are presented as mean ± SEM. Statistical significance of the differences is determined by two-way ANOVA followed by Tuckey’s *post hoc* test. *P*-values of the significances of the two factors and their interaction, analyzed by ANOVA, are provided in the table under the graphs. On the graphs, different p-values are shown by asterisks as described in Materials and Methods under Statistical Analysis section.

Since thiamine deficiency inverted the sensitivity of A549^WT^ and A549^p21–^ cells to cisplatin treatment (Figures 1E,F), the metabolic response of the cells to thiamine deficiency has been studied (Figure 4). The thiamine-deficient A549^WT^ cells show no significant alterations in the levels of GDH, GOT2 or holo-OGDHC (Figures 4A–C). However, the A549^WT^ cells adapt to thiamine deficiency employing the associated enzymatic network. The activity of NAD^+^-dependent IDH, providing 2-oxoglutarate for the OGDHC in the TCA cycle, potentially competing with 2-oxoglutarate from glutamate, is duplicated (Figure 4E). The activity of MDH is not changed by thiamine deficiency (Figure 4F). The activities of NADPH-producing ME and IDH are also duplicated in the thiamine-deficient vs. control A549^WT^ cells (Figures 4G,H).

The p21 knockdown strongly influences the adaptation of A549^p21–^ cells to thiamine deficiency. Although the activities of GDH, OGDHC and NAD^+^-dependent IDH are not changed by thiamine deficiency in A549^p21–^ cells, the level of GOT2 is increased 5-fold in the A549^p21–^ cells (Figures 4A–E), along with a small, but statistically significant upregulation of NADP^+^-dependent ME and IDH. MDH activity does not respond to thiamine deficiency in A549^p21–^, similar to A549^WT^ cells (Figures 4F–H). Overall, the thiamine-deficient A549^p21–^ cells compensate for their lower, compared to the thiamine-deficient A549^WT^ cells, activation of the producers of NAD(P)H by glutamate transamination. The inability of the thiamine-deficient A549^p21–^ cells to upregulate the NAD(P)H producers as strongly as the thiamine-deficient A549^WT^ do (Figures 4E,G,H), coincides with a higher sensitivity to the cisplatin damage of the former, compared to the latter cells (Figure 1F).

The different metabolic responses of A549^WT^ and A549^p21–^ cells to the thiamine deficiency correspond to their ability to upregulate p53 under the lack of thiamine. That is, only A549^WT^, but not A549^p21–^ cells, significantly increase expression of p53 upon thiamine deficiency (Figure 4I). In contrast, upon the cisplatin exposure, it is A549^p21–^, but not A549^WT^ cells, that upregulate p53 (Figure 3I). However, independent of the settings inducing p53 in either A549^WT^ and A549^p21–^ cells, those cells which are able to upregulate p53, are more resistant to the cisplatin action than those which do not.

### p53-Dependent Regulation of the 2-Oxoglutarate/Glutamate Interconversion Upon Changes in Cellular Thiamine Status Is Different in A549^WT^ and A549^p21–^ Cells

The p53/p21-dependent changes in the studied metabolic enzymes in A549 cells upon thiamine deficiency (Figure 4) or cisplatin exposure (Figure 3) suggest that the metabolic control by p53 is changed by p21 knockdown, and the changes are involved with cisplatin sensitivity of the cells (Figures 2E,F). To further characterize the p53-dependent metabolic control, linked to the different thiamine status of A549 cells, we have correlated the levels of enzymes and p53, assayed upon variations in cellular thiamine status. The varied pools of intracellular thiamine compounds were achieved by growing the cells in the thiamine-free, standard or supplemented with a high concentration of ThDP (5 mM) media. The major intracellular derivative of thiamine, ThDP, has been added instead of thiamine in view of ThDP involvement in regulation of p53 transcriptional activity (McLure et al., 2004). Given published evidence on transport of ThDP by cellular membrane transporters SLC19A1 (Zhao et al., 2001; Zhao and Goldman, 2013) and SLC44A4 (Nabokina et al., 2014), this experimental design may overcome complex regulation of ThDP synthesis in cancer cells (Jonus et al., 2018), being more straightforward to extend the interval of intracellular ThDP content. Expression of SLC19A1 is documented in A549 cells (Kawami et al., 2015), as is the expression of choline transporters-like CTL/SLC44 proteins (Inazu, 2014) sharing the same family with the ThDP-transporting SLC44A4. Although the exact molecular nature of the ThDP transporter in A549 cells requires further studies, the accumulation of intracellular ThDP under the employed conditions has been shown in our previous work (Bunik et al., 2020b) by detecting increased saturation of intramitochondrial 2-oxoglutarate dehydrogenase with ThDP after incubation of A549 cells in the medium with 5 mM ThDP.

**FIGURE 5:**
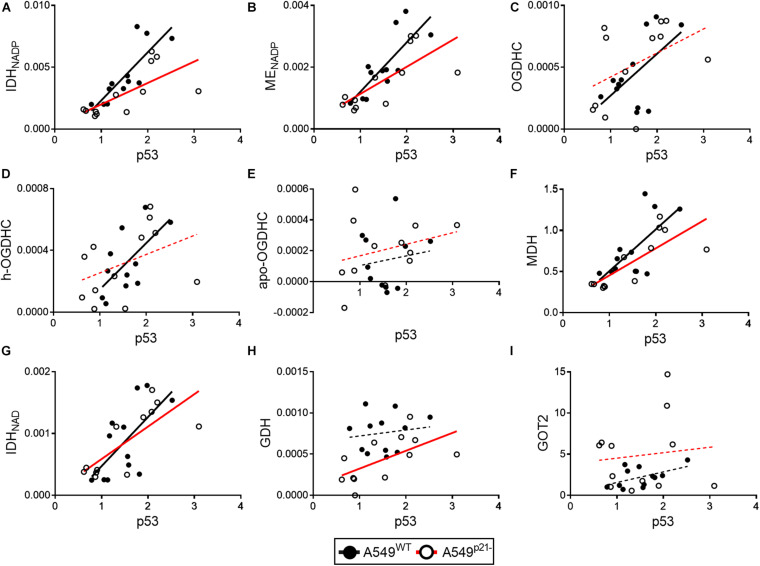
Changes in correlations between the levels of p53 and studied enzymes in A549^WT^ and A549^p21–^ cells. Levels of p53 protein (horizontal axes) are correlated to activities or expression of the enzymes, indicated on the graphs (vertical axes). **(A–H)** Enzyme activities are shown in μmol⋅ min^–1^⋅ mg^–1^; protein levels of the mitochondrial aspartate aminotransferase (GOT2) **(I)** and p53 are indicated in arbitrary units. OGDHC—total OGDHC activity, h-OGDHC—activity of holo-OGDHC, assayed at endogenous level of ThDP, apo-OGDHC—ThDP-free (not active) OGDHC, determined as the difference between the activities of the total and holo-OGDHC. Linear regressions of Pearson’s correlations are presented by black (A549^WT^) or red (A549^p21–^) lines, which are solid (*p* < 0.05) or dashed (*p* > 0.05) depending on the *P*-values of the correlation significance. Each point represents a separate cell culture from the three independent experiments.

In A549^WT^ cells, the assayed activities of the NADP^+^-dependent IDH and ME, contributed by the known p53 targets IDH1, IDH2 (Kil et al., 2006), ME1 and ME2 (Jiang et al., 2013; Woo et al., 2016), show strong positive correlations (*p* < 0.01) with p53 expression (Figures 5A,B and Table 1). Activities of OGDHC (total and holo), MDH and NAD^+^-dependent IDH3 (encoded by *IDH3A*, *IDH3B* and *IDH3G*) show less significant and moderate correlations with p53 expression (Figures 5C–G and Table 1). The levels of GDH and GOT2 do not correlate with p53 expression (Figures 5H,I). The correlations between the enzymes in A549^WT^ cells (Table 1, top right corner) favor existence of the common p53-dependent control of the dehydrogenases of isocitrate and malate, which show significant correlations to each other, and OGDHC, correlating with most of the studied enzymes and p53. In contrast, the levels of GDH and GOT2, which do not correlate with p53 expression, show low level of interdependence with other enzymes.

**TABLE 1:** Correlations between the levels of p53 and studied enzymes in A549^WT^ (top right corner) and A549^p21–^ (bottom left corner) cell lines at varied thiamine/ThDP levels.

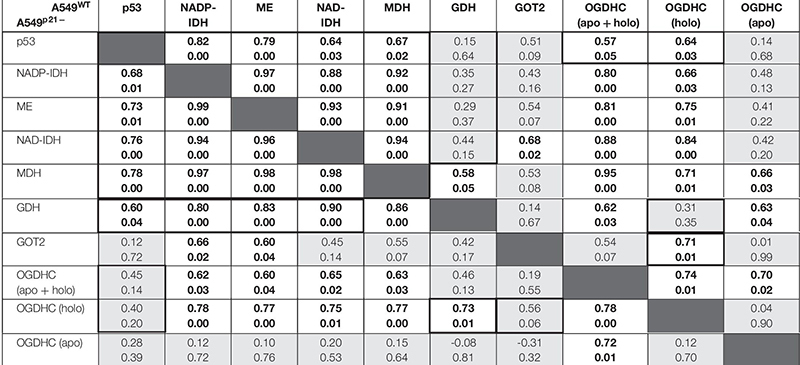

**Each cell of the table shows the corresponding Pearson correlation coefficients (above) and P-values (below). The data in bold depict significant correlations (p < 0.05), the cells in light-gray correspond to non-significant correlations (p > 0.05). The framed parts show the permanent and variable parts of the correlations in the two cell types. All data successfully passed the D’Agostino and Pearson omnibus normality test.**

In A549^p21–^ cells, the positive correlations between the p53 expression and activities of the NADP^+^-dependent IDH and ME remain high and significant (Figures 5A,B), while those between p53 and MDH and NAD^+^-dependent IDH3 even increase (Table 1, bottom left corner). However, p21 knockdown switches the correlations of the OGDHC and GDH activities with p53 expression. Activity of GDH, but not that of OGDHC, correlates with expression of p53 in A549^p21–^ cells. The opposite is observed in A549^WT^ cells, where activity of GDH does not correlate with p53, but that of OGDHC does. Moreover, activities of the TCA cycle enzymes OGDHC and NAD^+^-dependent IDH form mutually correlated entity with GOT2 in A549^WT^ cells, and with GDH in A549^p21–^ cells (Table 1). The switch is added by changed interdependences of OGDHC, GDH, and GOT2 with other enzymes.

Thus, upon variation of cellular level of the major thiamine derivative ThDP, p21 knockdown of A549 cells switches the p53 dependence of the activities of OGDHC and GDH, regulating the glutamate partitioning between transamination or oxidation in the TCA cycle.

## Discussion

In this work, we reveal metabolic changes, induced by p21 knockdown and/or thiamine deficiency, which correlate with different cisplatin effects on the viability of the non-small cell lung adenocarcinoma cell line A549. This cell line is used as a model of the top killer cancer, whose deadly nature is tightly linked to developing chemoresistance dependent on p53/p21 axes. Besides, specific metabolic features and molecular mechanisms supporting these features, have been previously characterized in A549 cells to the extent, enabling experimental design of our current study. In particular, these cells are known to rely on the function of a ThDP-dependent OGDHC and malate-aspartate shuttle (Allen et al., 2016; Bunik et al., 2016), both involved with the p53/p21 axes, particularly affecting the ThDP saturation of OGDHC (Zhou et al., 2015; Bunik et al., 2020b). Thus, we focus on the enzymes of TCA cycle and malate-aspartate shuttle, which are the pathways employing common metabolites to generate mitochondrial NADH oxidized in the electron transport chain. Our results reveal the metabolic patterns which adapt A549 cells to the cisplatin action, p21 knockdown and thiamine deficiency. Similarities in the metabolic responses to these factors underlie their interactive action upon combinations of the factors. This is shown by the thiamine-deficiency-switched sensitivity of A549^WT^ and A549^p21–^ cells to cisplatin (Figures 1E,F) or by the ANOVA analysis of the interaction of p21 knockdown with metabolic response of A549 cells to cisplatin or thiamine deficiency (Figures 3, 4). Essential components of metabolic network, which define such interactions, are involved in a switch between the glutamate oxidation by GDH and transamination by GOT2, linked to functions of the dehydrogenases transforming or producing the metabolites in common with the GDH and GOT reactions, i.e., 2-oxoglutarate, malate, oxaloacetate.

Our finding that p21 knockdown decreases the level of GDH increasing that of the malate-aspartate shuttle enzyme GOT2 (Figure 2), reciprocates earlier data that perturbed dicarboxylates shuttling between mitochondria and cytosol reduces p21 levels (Zhou et al., 2015). Cisplatin exposure of A549^WT^ cells has the same effect on GDH and GOT2 as p21 knockdown, which underlies strong interaction of the metabolic effects of the cisplatin exposure and p21 knockdown (Figure 3). Although the GDH decrease and GOT2 increase in the thiamine-deficient A549^WT^ cells are not statistically significant, these cells decrease the glutamate oxidation to 2-oxoglutarate by increasing competitive production of 2-oxoglutarate by NAD^+^-dependent IDH of the TCA cycle (Figure 4). In this regard, the decreased sensitivity of the thiamine-deficient vs. control A549^WT^ cells to cisplatin (Figures 1E,F) may be supported by combining the mechanisms of cellular adaptations to thiamine deficiency and cisplatin, such as activation of the 2-oxoglutarate production in the TCA cycle and decreased glutamate oxidation. Thus, the regulation of the glutamate partitioning between the GDH and GOT2 reactions, observed in our work upon a number of challenges, such as perturbed p53/p21 regulation, cisplatin exposure or thiamine deficiency, represent a rather universal mechanism of cellular adaptation. As demonstrated in independent studies, the glutamate accumulation is accompanied by a decrease in ROS, in accordance with the glutamate role in biosynthesis of the ROS scavenger glutathione, both *per se* and through the exchange with extracellular cystine (Bunik et al., 2016; Hiyama et al., 2018). Hence, the observed changes in the glutamate metabolism in A549 cells may support the known upregulation of glutathione biosynthesis in the cisplatin-resistant lung adenocarcinoma cell lines (Hiyama et al., 2018). The elevated dependence of the cisplatin-resistant cells on glutamine, which is needed for glutathione biosynthesis, has been linked to their increased mitochondrial metabolism resulting in increased ROS level (Wangpaichitr et al., 2017; Okazaki et al., 2019). Our observation of the cisplatin-induced activation of the TCA-cycle-limiting OGDHC along with the upregulation of the IDH and MDH reactions producing the TCA cycle metabolites 2-oxoglutarate and oxaloacetate (Figure 3), also demonstrates activation of mitochondrial metabolism by cisplatin in A549^WT^ cells. Simultaneously, cisplatin treatment of A549^WT^ decreases glutamate oxidation. Thus, the metabolic effects induced by cisplatin in A549^WT^ cells, may manifest their adaptations eventually leading to development of cisplatin resistance.

Although different cancer cells are known to vary in the studied metabolic and regulatory features of A549 cells, our study highlights principal components of the metabolic and regulatory network, supporting cellular adaptations to thiamine deficiency and cisplatin exposure. Previous studies (Bunik et al., 2015; Allen et al., 2016; Bunik et al., 2016) indicate that unraveling such components and their combinations in specific cell types is essential for our ability to predict sensitivity of different cells to external factors, such as nutrients availability and drugs exposure, using, in particular, high-throughput “omics” data. Our current results on A549 cells are therefore in good accord to independent studies of other cell types, where the related pathways have been considered. For instance, targeting the cystine/glutamate exchanger SLC7A11 increases cisplatin sensitivity in many studies (Drayton et al., 2014; Okazaki et al., 2019). The glutaminolysis-related genes, including ASCT2 and GDH-encoding *GLUD*, are suggested as biomarkers of the efficacy of SLC7A11 (xCT)-targeted therapy for heterogeneous head and neck squamous cell carcinoma tumors (Okazaki et al., 2019). Ferroptotic death, dependent on inhibited function of SLC7A11, is considered as a major mechanism directing the cisplatin-resistant cells to death (Roh et al., 2016), although the role of additional pathways, particularly those supported by NAD^+^ and lactate dehydrogenase A (Wangpaichitr et al., 2017), has also been shown. Remarkably, acetylation of p53 at K98 induces the ferroptosis upon impairment of SLC7A11 (Wang et al., 2016), while the thiamine and OGDHC involvement with glutamate metabolism employs the regulation of posttranslational acetylations of proteins, particularly p53 and GDH (Mkrtchyan et al., 2015; Aleshin et al., 2020; Boyko et al., 2021).

Permanent endoplasmic reticulum stress has been shown to contribute to mechanisms of the cisplatin resistance (Bahar et al., 2020; Chen et al., 2020). In neurons, such stress is known to be induced by thiamine deficiency (Wang et al., 2007; Liu et al., 2017; Xu et al., 2019b). Unlike the ROS production, strongly dependent on the contributing pathways whose expression is different in the normal and cancer cells, the poor control of protein synthesis may be a common consequence of thiamine deficiency in the normal and cancer cells. Endoplasmic reticulum stress may explain reduced viability of the thiamine-deficient A549 cells despite their higher glutathione content (Bunik et al., 2020b). Pre-adaptation to such stress upon thiamine deficiency may contribute to the reversal of the antiproliferative action of cisplatin in the thiamine-deficient A549^WT^ cells (Figures 1E,F).

As a result, our work shows that the cisplatin effects on cellular viability are strongly influenced by thiamine deficiency. Because thiamine deficiency often occurs in oncopatients without its classical symptoms (Ahmad et al., 2016; Choi et al., 2016; Isenberg-Grzeda et al., 2016, 2017; Onishi et al., 2018; Yoshioka et al., 2020; Zhang et al., 2020), the blood thiamine levels should be determined in such patients, and potential interference of the thiamine hypovitaminosis with the anticancer treatments considered.

## Data Availability Statement

The raw data supporting the conclusions of this article will be made available by the authors, without undue reservation.

## Author Contributions

VA performed the acquisition, validation and formal analysis of the data, and participated in writing the manuscript draft. XZ contributed to the project methodology and investigation. SK assayed p21. AK and VB performed funding acquisition, project administration and conceptualization. VB wrote the manuscript. All co-authors provided final approval of the version to be published, and agreed to be accountable for all aspects of the work.

## Conflict of Interest

The authors declare that the research was conducted in the absence of any commercial or financial relationships that could be construed as a potential conflict of interest.

## Abbreviations

A549^WT^: control A549 cell line
A549^*p21*–^: p21-knockdown A549 cell line
GDH: glutamate dehydrogenase
OGDHC: 2-oxoglutarate dehydrogenase complex
IDH: isocitrate dehydrogenase
MDH: malate dehydrogenase
ME: malic enzyme
GOT2: mitochondrial glutamic-oxaloacetic transaminase 2
ThDP: thiamine diphosphate.
